# A Dialog on the First 20 Years of PML Research and the Next 20 Ahead

**DOI:** 10.3389/fonc.2014.00023

**Published:** 2014-02-10

**Authors:** Rosa Bernardi, Pier Paolo Pandolfi

**Affiliations:** ^1^Division of Molecular Oncology, San Raffaele Scientific Institute, Milano, Italy; ^2^Cancer Research Institute, Beth Israel Deaconess Cancer Center, Department of Medicine and Pathology, Beth Israel Deaconess Medical Center, Harvard Medical School, Boston, MA, USA

**Keywords:** leukemia, tumor suppressor, PML, nuclear bodies, apoptosis

## Abstract

This introductory article has been written in the form of a conversation between Pier Paolo Pandolfi, Director of the Cancer Center of Beth Israel Deaconess Medical Center in Boston, and Rosa Bernardi, a former post-doctoral fellow in the laboratory of Dr. Pandolfi, now principal investigator at San Raffaele Scientific Institute in Milan, Italy. We have chosen this atypical review format because we want to offer to our readers a more direct and personal perspective on the first 20 years of research over the promyelocytic leukemia gene. This article begins as an interview, but soon transforms into a dialog where we exchange our thoughts on a number of issues around the past, present, and future research over the biology of PML. We were particularly keen on emphasizing the aspects that we find most interesting or challenging, therefore, we warn our readers that this will not be a comprehensive essay but rather a very personal view of what has been, is, and will be exciting and interesting in the PML world, in our opinion.

## Introduction

**Figure 1:**
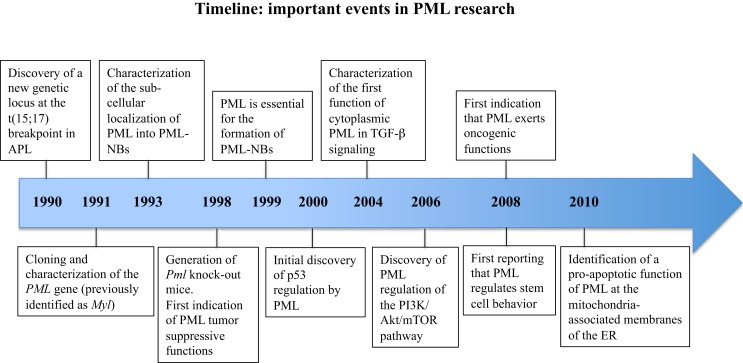
**Timeline of the milestone discoveries on PML**.

The promyelocytic leukemia gene, *PML* was originally discovered because of its involvement in acute promyelocytic leukemia (APL) (1–4), and was later shown to encode a homo-multimeric protein with predominant nuclear localization, wide tissue expression, and prevalent tumor suppressive functions, both in hematological and solid malignancies (Figure 1).

Twenty years of intense research activity carried out by a number of laboratories and prompted by the generation of *Pml* knock-out (KO) mice (5), have led to the description of PML as a widely multifaceted protein that regulates many aspects of normal physiology and pathology (Figure 1). Complexity is the term that best defines PML at many levels: the *PML* gene undergoes multiple alternative splicing processes and generates a variety of PML mRNA species and protein isoforms; PML isoforms differ in a number of features, from protein interaction capabilities and sub-cellular localization to specific functions within the cell; PML functions are often subtle, and unveiled only upon specific experimental challenges; the biochemistry of PML interactions is particularly difficult to tackle because of the insolubility of PML protein complexes. Regardless of these difficulties, or perhaps precisely because of them, the study of PML functions has constantly attracted the attention of many researchers. Such keen interest had led over the years to the incessant addition of new levels of complexity to the biology of PML. Reconciling everything that is currently known into a unifying working model is at the present time impossible, and probably unnecessary. For this reason, we have chosen to freely discuss about what we find most interesting and promising about research on PML, beginning with a very personal account on how it all started.

## A Dialog on PML

Bernardi: Pier Paolo, in 1991 as a medical student to start with, and a freshly graduated post-doctoral fellow in the laboratory of Pier Giuseppe Pelicci (in those times leading his laboratory in Perugia, now directing the Department of Experimental Oncology at the European Institute of Oncology in Milan, Italy), you cloned and characterized the *PML* transcript and its aberrant version *PML–RAR*α. This new genetic locus had been identified a year earlier at the breakpoint of *t*(15;17) translocation along with the retinoic acid receptor α gene (*RAR*α) in APL through the work of Letizia Longo and yourself in the Pelicci laboratory, as well as by other groups (6–8). You told me once that cloning and characterizing the *PML* gene was a tough race; one of those exciting and hysterical moments in science when you know that like you other people, in other laboratories, are working day and night to publish their results first. Out of this race, within a time frame of a few months, four papers described the characterization of the *PML* gene [renamed *PML* after having been identified as *Myl* (6)] and the PML–RARα fusion protein (1–4). This project must have changed your life, and unquestionably shaped your career: out of this line of research came your first KO mouse and your longstanding interest for leukemia gene rearrangements, which later widened to include a larger spectrum of projects on the genetic bases of cancer. What do you recall of those early days? What have been the most exciting or frustrating moments?

Pandolfi: In those times, I had just begun working in a laboratory as a medical student and as a summer student after a few years of thrilling philosophy studies at the University of Rome, and everything about science and research was very novel and exciting. Furthermore, the idea of cancer oncogenes really intrigued me. The opportunity of being involved in such an ambitious project, the cloning of a new cancer gene was from the get go pure adrenaline. However, I will never forget when we obtained the first PML mRNA sequence and attempted to find an open reading frame that would mean something structurally: nothing! It was a big disappointment. Now we know that PML has an RING domain embedded into an RBCC or TRIM structure (9, 10), but at that time RING fingers were not characterized. We were therefore really in the dark. But the first antibody raised to recognize PML gave the whole APL field some hope. We were seeing nuclear dots, what we now call in jargon the PML-nuclear bodies [PML-NBs; Ref. (11)]. This was indeed intriguing. But the real question that was not making me sleep at night was whether PML was an oncogene, or else, and whether it would be important in the control of hemopoiesis.

Bernardi: On my side, what I found most remarkable when I begun working on PML is the beauty of its sub-cellular localization into the PML-NBs. These distinctive macromolecular structures had been known to exist since the 60s (12), but only in 1993 they were identified as sites of accumulation of PML, being renamed at that point PML-nuclear bodies (13). We now know a lot of things about PML-NBs: they are donut-shaped, with a diameter of 0.2–1.0 μm, there is an average of 5–15 of them per cell nucleus, and they are formed by a ring of proteins surrounding an empty space [sometimes occupied by microgranular material proposed to be ribonucleoproteins (12), with DNA and RNA localizing at the periphery of the ring (14, 15)]. However, it seems to me that even though we have learnt a lot about their biochemistry and function, much still remain to be uncovered. For instance, although we keep referring to them as PML-NBs, as if they were homogeneous structures, in reality they probably are not: they vary in dynamics, biochemistry, and structure in the different phases of the cell cycle [with mitotic PML aggregates being so peculiar to have been termed mitotic accumulations of PML protein, or MAPPs (16)], in different cell types (i.e., embryonic stem cells versus cell lines), and upon different types of cellular stresses, such as heat shock or inhibition of transcription (12). However, besides a general recognition that there may be different types of PML-NBs, a precise characterization of their specific structure and function is far from being revealed.

I am also wondering whether a better characterization of PML protein isoforms may help us to better characterize different types of PML-NBs. Indeed, one other striking feature of PML is the high number of alternatively spliced mRNA molecules and protein isoforms that are generated from the *PML* gene. All PML isoforms contain the N-terminal homo-multimerization domain [termed RBCC or TRIM (9, 10)] that allows formation of high order molecular structures, but differ in their C-terminal regions (10), and when expressed individually in *Pml* null cells form morphologically and dynamically different PML-NBs (17, 18). This suggests that PML isoforms may play different roles in the formation and maintenance of PML-NBs. However, in the absence of a comprehensive mapping of the relative expression of PML isoforms and the relative composition of PML-NBs in different cell conditions, the biological relevance of PML isoforms’ specificity and their regulation of PML-NBs still remains to be established. I think that this is a very interesting field of research, where we should invest more; especially considering that specific PML isoforms have been suggested to have exclusive functions (19).

Pandolfi: Talking about PML isoforms and isoform specificity, what has been overlooked for a while and only recently has started to be seriously investigated is the role of cytoplasmic PML isoforms and functions. Although aggregates of PML are visualized mostly in the nucleus, we have known for a long time that a number of PML splicing isoforms come into a cytoplasmic flavor through exclusion of exon 6 (that contains a nuclear localization signal) (10–19). Also PMLI, the most abundant isoform, is both nuclear and cytoplasmic due to a nuclear export signal in its C-terminus, and even in the nucleus it is probably most abundant in the nucleoplasm rather that in the bright PML-NBs (19). To me, the identification of novel functions of PML in the cytoplasm has been among the most exciting recent advances of PML research. Interestingly, similar to what we see in the nucleus, also in the cytoplasm PML appears to be involved in a multitude of molecular functions: it activates TGF-β signaling by promoting the association of TGF-β receptors with SARA and Smad proteins at the early endosome (20); it facilitates transfer of calcium to the mitochondria and apoptosis by localizing at the mitochondria-associated membranes of the endoplasmic reticulum (21); it interacts with and suppresses the function of the M2 isoform of pyruvate kinase (PKM2) thus suppressing tumor metabolism (22), even though these findings are preliminary; and it regulates a number of anti-viral responses (23).

Going back to the idea that different PML isoforms form different PML-NBs, one could imagine that function multiplicity in the cytoplasm may also be explained by the presence of different cytoplasmic PML isoforms, although in the absence of a more precise characterization of isoforms’ representation, this can only be speculated at the moment. Alternatively, the specificity of PML functions in the cytoplasm could be caused by its accumulation into specific structures, such as endosomes or endoplasmic reticulum, rather than by isoform specificity. In this respect, we had observed that PML-induced stimulation of TGF-β signaling could occur also upon expression of a truncated PML mutant expressing only the N-terminal RBCC domain, and lacking any C-terminal region in *Pml* null cells (20). In my view, a systematic *in vivo* analysis in isoform-specific KO models is very much needed and critically important; similarly important is the generation of mouse mutant in which the PML-NBs could be visualized in their *in vivo* dynamics. We are actively working on this.

Bernardi: With regard to the second point that you are making, the idea that the regulation of PML functions may depend considerably on its sub-cellular localization, this is further substantiated by the notion that dynamic behaviors of PML have been described also within the constraints of the cell nucleus. Indeed, I was quite puzzled from the very first experiment that I conducted in your lab, as I was studying the regulation of p53 by PML in conditions of DNA damage, because I kept observing that upon specific DNA damaging conditions, PML appeared to localize to an external shell surrounding the nucleolus (24). Previous studies had already described the localization of PML to the nucleolus upon inhibition of the proteasome (25). Interestingly, however, later studies by the group of Hugues de Thé demonstrated that localization of all PML isoforms to the nucleolus, or to aberrant nucleolar structures upon different kinds of cellular stress, is probably mediated by PMLI, the most abundant PML isoform and the only one that contains a nucleolar localization domain hidden in its C-terminus (26). This takes us back to the issue of isoform specificity.

In any case, regardless of the specificity of PML isoforms, as suggested by the group of Hugues de Thé, the functional interplay between PML and the nucleolus must be quite important as it recurs in a number of circumstances in which PML plays an important role, ranging from the response to DNA damage to cell senescence. Also, the C-terminal region of PMLI, which contains the nucleolar-targeting domain, is the most evolutionarily conserved (26).

Pandolfi: It is well possible that the nucleolar localization of PML might be crucial to exert some of its functions. Certainly though, not all that PML does go through the nucleolus. And more generally, what remains difficult to summarize for a general audience is what exactly PML does in these various cellular sites, and whether it exerts a specific enzymatic activity. The latter still remains the key and most challenging question in the field.

From a historical perspective, research over the function of PML has been greatly stimulated by the generation of *Pml* KO mice (5), and the availability of primary cells lacking *Pml* expression. From those early days on, through the work of many laboratories all over the world, I think that the basic message that we have learnt is that PML is a widely multifaceted protein that regulates many important aspects of normal physiology and pathology. However, PML functions are often subtle and unveiled only upon specific experimental challenges. I still vividly remember the initial disappointment when we discovered that *Pml* was dispensable for embryonic life. I had moved to London to KO *Pml* in the mouse after having read the seminal paper from Mario Capecchi and colleagues describing the KO of the proto-oncogene int-2 [FGF3 (27)]. Those were early days, and the *Pml* KO mouse was one of the first ever generated in Europe. After so much work to make the *Pml* KO mouse, we were imagining the most severe of phenotypes, and yet this was not the case: apparently the mice were fine. In retrospect, though, we got our revenge in the years to follow. Since then, many exiting functions of *Pml* were subsequently unraveled by our group and others because of the *Pml* KO model.

Among the most important functions of PML, relevant to its tumor suppressive activity is its ability to positively regulate the function of p53 and family members, and negatively the PI3K pathway, at multiple levels. The regulation of p53 by PML was one of the first functions of PML to be dissected in molecular detail (28–30), and over the years has been the most extensively validated: literature over the cross-regulation of PML and p53 and its family members has reached the two-digit figure, with approximately 20 independently published studies. The take home message is that PML controls the expression and activity of p53 and family members through multiple mechanisms, including facilitating a number of post-translational modifications of p53 and interacting with many factors that in turn regulate p53, p73, and p63 activity (31–33). Recently, two independent papers confirmed that PML and p53 also functionally interact *in vivo*, as loss of *Pml* contributes significant alternations in tumor phenotypes in two mouse models of p53-induced tumorigenesis (34, 35).

Bernardi: While I agree that this function of PML is the most validated, I find exciting that new functions of PML are still emerging, and I think that these new aspects of PML biology will be greatly investigated in the future. I am referring to the role of PML in stem cell biology and metabolism, which also relates to regulating the PI3K pathway, as you mentioned. Indications that PML regulates the biology of stem cells have come recently from two elegant and unrelated studies in which the function of PML was investigated in leukemia and hematopoietic stem cells (36), and in neural stem cells (37). In the first paper, PML was described as an essential regulator of stem cell maintenance, both in normal hematopoiesis and in chronic myeloid leukemia, as deletion of *Pml* in KO mice led to loss of quiescence and exhaustion of hematopoietic and leukemia stem cells (36). Interestingly, inhibition of Pml by arsenic trioxide disrupted maintenance of leukemia initiating cells thus sensitizing leukemia to treatment with other anti-leukemia agents (36). In the second paper, a thorough analysis of the developing brain of *Pml* KO mice revealed that PML suppresses proliferation and associates with the immature state of neural progenitors by promoting cell cycle arrest (37).

As per the mechanisms involved in PML-regulated maintenance of stem cell potential, an association of *Pml* loss with high mTOR activity, in turn known to drive stem cell exhaustion, was initially observed in hematopoietic stem cells (36). This observation further confirmed previous work where we had described PML as a negative regulator of mTOR activity in hypoxic conditions, thus leading to inhibition of hypoxia-inducible factors signaling and tumor neo-angiogenesis (38). I think that this is also very much coherent with the fact that the hematopoietic stem cell niche is known to be a hypoxic environment. More recently, however, it was also shown that in hematopoietic stem cells PML stimulates fatty acid metabolism (39), which in turn regulates stem cell maintenance and asymmetric stem cell division at the expense of symmetric cell commitment (39). In the context of neural stem cells, PML was implicated in a different mechanism of action, in line with its ability to maintain quiescence: pRb-mediated regulation of cell cycle entry (37).

In summary, although a role for PML in regulating stem cell-ness is emerging, mechanistically it is possible that PML regulates stem cell maintenance through different mechanisms in different tissues. In this respect, it should be mentioned that PML was recently shown to also promote the expression of the stem cell factor Oct4 in proliferating embryonic cells by regulating an open chromatin state in its promoter (40).

Pandolfi: You rightly mention metabolism as the other emerging aspect of PML biology. The regulation of metabolism by PML further complicates our simplistic perception of PML as a “good guy”: the tumor suppressor for any stressful cellular condition. Over the years, this notion had been very reassuring, and as you know we are always reassured by simple models. However, PML, which had been considered a tumor suppressor gene for over 20 years, is now found to exert oncogenic functions in specific tumor contexts precisely because of its metabolic role. We came to the understanding that PML might also behave as an oncogene while we were further investigating its role in regulating metabolism (39). We had already shown in several important studies that PML regulates at multiple levels a very important proto-oncogenic pathway, the PI3K/PTEN/AKT/mTOR signal transduction pathway, which is also critical in metabolism and metabolic homeostasis. We had found that PML opposes this pathway at multiple levels: by favoring PTEN proper nuclear cytosolic shuttling though its ability to oppose HAUSP deubiquitilation (41); by inactivating nuclear Akt through dephosphorylation into the PML-NBs (42), and by regulating nuclear accumulation and activity of mTOR in conditions of hypoxia, thus impinging onto hypoxia-responsive pathways (38).

More recently, however, we found that PML exerts another important metabolic function by acting as a potent activator of PPAR signaling and positively regulating fatty acid oxidation (39). While this function of PML supports asymmetric cell division in normal hematopoietic stem cells (39), it also inhibits cell death by anoikis hence favoring cell survival in breast cancer cells (39). Therefore, together with our previous identification of PML as a protective agents against leukemia stem cell exhaustion (36), these new data reinforce the concept that in specific cell contexts PML may also play oncogenic functions besides its best-known tumor suppressive activities. Implications of these new data have been recently discussed in two dedicated reviews (43, 44). Interestingly, another study indicates that in *Pml* KO tissues a variety of metabolic pathways are tissue-specifically de-regulated with respect to wt animals, such as for instance glucose metabolism and AMPK activation, thus suggesting that the role of PML in regulating metabolism may go beyond the regulation of fatty acid oxidation (45).

Bernardi: Certainly, the idea that PML has pro-oncogenic functions in specific contexts is intriguing. Dual activities are not unprecedented for oncogenes and tumor suppressors alike, and it is plausible that different outcomes of PML expression may be dictated by cell specific contexts. Indeed, although many studies have documented loss of expression of PML in tumors of different histological origin, more recently high expression of PML has been reported in triple-negative breast cancer and chronic myeloid leukemia (36, 39). A future challenge will be to understand what are the discriminating genetic co-factors of PML oncogenic or tumor suppressive functions and which other, if any, tumor type might benefit of PML tumor-promoting functions.

In closing, tell me three aspects that excite you the most regarding PML research in the next 20 years.

Pandolfi: I would say: (i) PML regulation and function; (ii) PML enzymatic activity; and (iii) implication for therapy, and not necessarily in order of importance. Regarding PML regulation, we have mentioned PML isoforms, but I think that there is a lot to be learned by studying the mechanisms or post-transcriptional regulation of PML, and undoubtedly, additional functions of PML will be uncovered in the future. Importantly, I think that we are still scratching our heads concerning whether or not PML exerts a distinct enzymatic activity. While I am excited by the essential role that PML plays in the formation of the PML-NBs (11), I still hope that there’s an enzymatic function of PML that we have failed to discover thus far. A possible PML enzymatic activity, if any, will prove extremely instructive of PML research in the years to come. Lastly, I hope that collectively we will find pharmacological ways to modulate PML therapeutically for the treatment of cancer and other disorders. This proved already critical to the eradication of APL. We should never forget that one of the main reasons why arsenic trioxide has led to APL cure is because it can target PML-RAR and also PML for degradation (39, 46). I therefore think that in the years to come PML research will pave the way to new discoveries and therapies for cancer and other diseases. So, we will keep actively working on PML and you Rosa should do the same in your lab!

Bernardi: I sure will.

## Conflict of Interest Statement

The authors declare that the research was conducted in the absence of any commercial or financial relationships that could be construed as a potential conflict of interest.
